# Lateral Access Spine Surgery following Liver Transplantation: A Report of Two Cases

**DOI:** 10.1155/2021/5581952

**Published:** 2021-03-13

**Authors:** Koji Akeda, Norihiko Takegami, Junichi Yamada, Masashi Kishiwada, Hiroyuki Sakurai, Shugo Mizuno, Shuji Isaji, Akihiro Sudo

**Affiliations:** ^1^Department of Orthopaedic Surgery, Mie University Graduate School of Medicine, Mie, Japan; ^2^Department of Hepatobiliary Pancreatic and Transplant Surgery, Mie University Graduate School of Medicine, Mie, Japan

## Abstract

With improving prognosis for recipients of liver transplantation, the necessity of medical care for musculoskeletal disorders, including spinal diseases, of these patients is also increasing. Only a few reports are available on cases of spine surgery following liver transplantation. Furthermore, a case of lateral access surgery following liver transplantation has not thus far been reported. The purpose of this study is to present the first two cases of patients treated with lateral access spine surgery following liver transplantation. *Case 1*. A 49-year-old female had received living donor liver transplantation (LT) for acute-on-chronic liver failure (ACLF) ten years prior to spine surgery. The patient underwent two levels of lateral lumbar interbody fusion (LLIF) followed by posterior lumbar interbody fusion (PLIF) for degenerative lumbar disease. Although neurological symptoms in the lower extremity improved, a liver disorder resulting from acetaminophen-induced hepatotoxicity occurred at an early stage following spine surgery. *Case 2*. A 66-year-old female had received living donor liver transplantation for hepatocellular carcinoma (HCC) six years prior to spine surgery. She underwent posterior instrumentation surgery followed by a T12 corpectomy using a wide-foot print expandable cage for T12 vertebral collapse. Following surgery, her weakened muscle strength in the lower extremities was significantly improved. Lateral access spine surgery for lumbar and thoracolumbar lesions can be successfully performed for patients following liver transplantation. However, careful follow-up should be performed for complications related to the function of the liver graft following spine surgery.

## 1. Introduction

Organ transplantation, including liver transplantation, has become more prevalent in recent years. Liver transplantation is the gold standard treatment for acute liver failure, end-stage liver disease, and primary hepatic malignancy [1]. Data on adult liver transplants performed in the United States showed that the number of liver transplants continued to increase over a recent decade (8082 patients in 2017, 7871 patients in 2016, and 6494 patients in 2007) [2]. With improvement in the long-term graft outcome of liver transplants, the medical management to improve activities of daily living (ADL) and quality of life (QOL) of the transplant recipient, including spine surgery [3], has become of great importance.

It has been reported that the transplant recipient has an increased risk of infection because of the necessity for immunosuppressant use [4]. The use of immunosuppressant and/or steroid after liver transplant can also result in the development of osteoporosis [5].

During the last decade in the field of spine surgery, lateral access spine surgery has remarkably evolved. The use of lateral lumbar interbody fusion (LLIF) through a retroperitoneal transpsoas lateral approach for the lumbar spine has progressively increased [6]. LLIF surgery can effectively restore disc height and foraminal height, thus, achieving indirect decompression of neural elements by the insertion of a large interbody cage into the intervertebral space [7, 8]. LLIF is useful in the treatment of degenerative spinal conditions with multiple studies reporting excellent improvement [6]. More recently, a minimally invasive retroperitoneal or retropleural lateral approach has also been applied for anterior stabilization with a mini-open corpectomy and cage replacement for patients with unstable spinal burst factures [9]. A recent clinical study showed that mini-open anterior corpectomy with wide-footprint expandable titanium vertebral body replacement is safe and effective for the functional recovery of patients with unstable burst fractures [10].

Only few reports are available on cases of spine surgery, especially spine fusion surgery using instrumentation, following liver transplantation [3, 11]. Furthermore, lateral access spine surgery following liver transplantation has thus far not been reported. This report describes the first two cases of lateral access spine surgery following liver transplantation.

## 2. Case Presentation

### 2.1. Case 1

**Figure 1:**
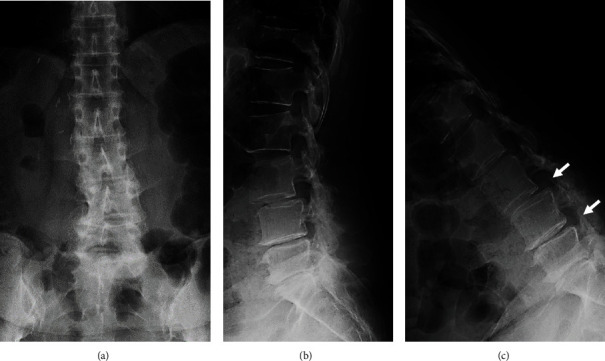
Preoperative lumbar spinal radiographs of a 49-year-old woman with degenerative lumbar disease. (a) anteroposterior view, (b) lateral view, and (c) lateral view (flexion position). Arrows indicate an intervertebral instability at L3/4 and L4/5.

**Figure 2:**
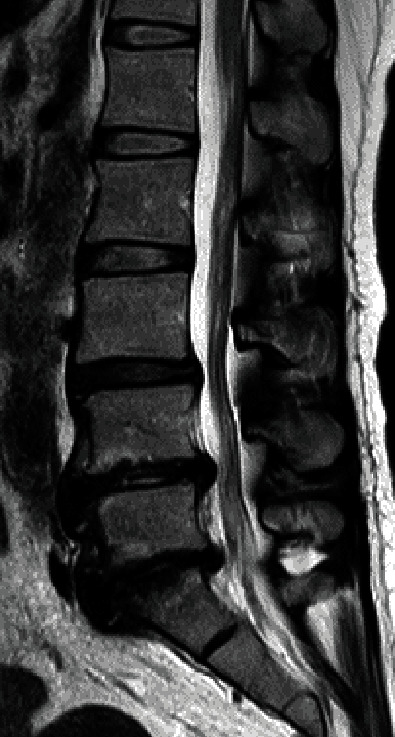
T2-weighted magnetic resonance imaging (MRI) of the lumbar spine of a 49-year-old woman with degenerative lumbar disease. Midsagittal section.

A 49-year-old female had received living donor liver transplantation (LT) for acute-on-chronic liver failure (ACLF) ten years prior to presentation (Table 1). The patient complained of low back pain and leg pain, which had not responded to drug therapy for two years. She presented with radicular pain from her buttock to her left leg and a significant decrease in walking capacity. The lateral lumbar radiograph showed an intervertebral instability [12] at L3/4 disc level with degenerative spondylolisthesis (sagittal translation +7.6 mm [% slip 22.4]; intervertebral angle [degree]: flexion -6.9/extension 3.2; segmental angulation [degree] 10.1) and L4/5 disc level (sagittal translation +1.4 mm [% slip 4.1]; intervertebral angle [degree]: flexion -9.2/extension 1.3; segmental angulation [degree] 10.5) (Figure 1). On magnetic resonance imaging (MRI), disc degeneration with disc bulging was found at the L3/L4, L4/L5, and L5/S1 disc levels (Figure 2). Spinal stenosis was found at the L3/L4 and L4/L5 disc levels (Figure 2), and foraminal stenosis was identified at the left L5/S1 foramen.

**Figure 3:**
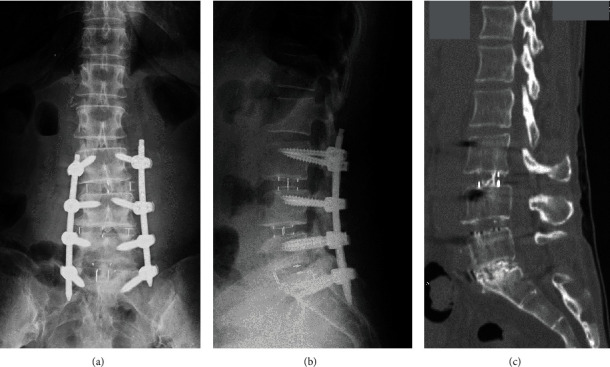
Postoperative radiographs and computed tomography (CT) image of the lumbar spine of a 49-year-old woman with degenerative lumbar disease. (a) anteroposterior view X-ray, (b) lateral view X-ray, and (c) sagittal CT image two years following surgery.

**Figure 4:**
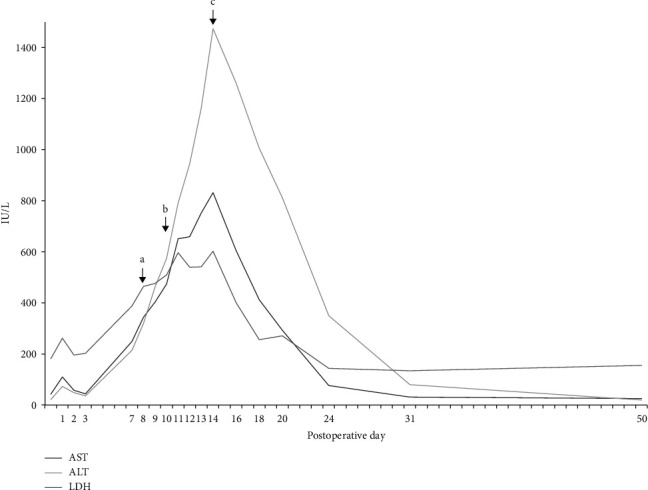
Temporal changes in serum levels of aspartate transaminase (AST), alanine transaminase (ALT), and lactate dehydrogenase (LDH). (a) all drugs, except for immunosuppressant drug and acetaminophen, were stopped. (b) Liver biopsy was performed. (c) The use of per-request medication (acetaminophen) was stopped.

The patient underwent lateral lumbar interbody fusion (LLIF) at L3/4 and L4/5, followed by posterior lumbar interbody fusion (PLIF) at L5/S1 and supplemental percutaneous posterior instrumentation (Figure 3). First, the LLIF procedure was performed as previously reported [13] with some modification. Briefly, the patient was placed in a left lateral decubitus position, with the skin incision (5 cm) between L3/4 and L4/5. Following the dissection of abdominal muscles, the retroperitoneal space was assessed by blunt dissection. The peritoneal content and ureter were safely mobilized anteriorly without adhesion with the surrounding tissue. The psoas muscle was identified, and intervertebral discs (L3/4 and L4/5) were exposed by dissecting the psoas muscle from 1 cm posterior from the anterior edge of the psoas muscle. After curettage of the disc tissues, appropriately-sized LLIF cages were inserted. A hydroxyapatite/collagen (HAp/Col) composite [14] (Refit®, HOYA, Japan) absorbed on autologous bone marrow aspirate (BMA) was packed into the cages. Next, a mini-open PLIF at the L5/S1 level was performed, pedicle screws were then inserted into the L3, L4, L5, and S1 levels percutaneously, and titanium alloy rods were placed (Figures 3(a) and 3(b)). The operation time was 336 minutes (lateral: 122 minutes, posterior: 214 minutes), and intraoperative blood loss was 109 ml. Postoperative intravenous antibiotic (Cefazolin: 1 g x 2/day) was administrated for seven days. Seven days following surgery, the levels of aspartate transaminase (AST) and alanine transaminase (ALT) increased to 249 IU/L and 214 IU/L, respectively (Figure 4(a)). The lactate dehydrogenase (LDH) level was also increased to 388 IU/L. Contrast computed tomography (CT) images identified neither blood flow disturbance nor morphological abnormality on the transplanted liver. Drug-induced hepatopathy or liver allograft rejection [15] was suspected. Although all drugs, except for immunosuppressant drug and acetaminophen, were stopped, serum levels of AST, ALT, and LDH continuously increased to 474 IU/L, 574 IU/L, and 510 IU/L at ten days postoperative, respectively (Figure 4(a)). Therefore, a liver biopsy was performed. A mild cellular rejection of liver tissue was diagnosed by histopathological examination; however, it was not equivalent to the clinical course of a severe liver disorder by liver allograft rejection. The serum level of these three enzymes rapidly increased and reached peak levels at 14 days following surgery (AST: 832 IU/L, ALT: 1473 IU/L, LDH: 602 IU/L) (Figure 4(c)). After prohibiting taking acetaminophen as needed, those serum levels gradually decreased and reached normal ranges 50 days following surgery (AST: 25 IU/L, ALT: 19 IU/L, LDH: 155 IU/L). Two months following surgery, leg symptoms, and low back pain were significantly improved. Sagittal CT images taken two years following surgery showed irregular bridging bone through the LLIF cage at L3/4 and L4/5 and robust bridging bone through the L5/S1 intervertebral space (Figure 3(c)). The patient could walk without recurrence of leg pain 2.5 years following surgery.

### 2.2. Case 2

**Figure 5:**
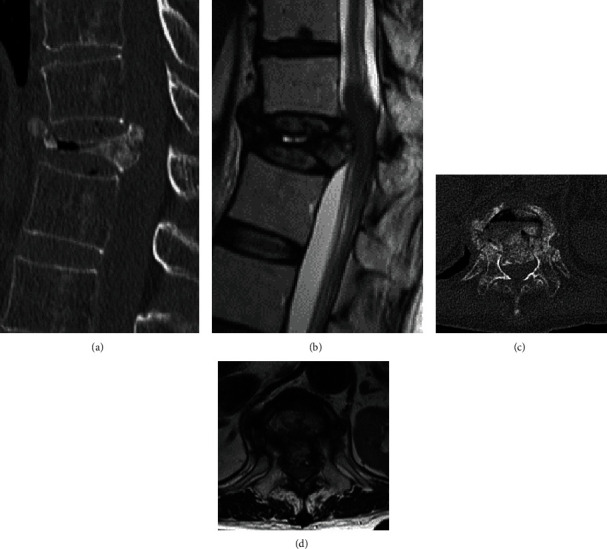
Preoperative thoracolumbar computed tomography (CT) images and magnetic resonance images (MRIs) of a 66-year-old woman with collapse of the 12^th^ thoracic (T12) vertebra. (a) sagittal CT image; (b) sagittal T2-weighted MRI; (c) axial CT image at the T12 vertebra; (d) Axial MRI image at the T12 vertebra.

A 66-year-old female, who had received a living donor LT for hepatocellular carcinoma (HCC) six years prior (Table 1), presented with back pain after a fall. Two months after the injury, she experienced a gradual decrease of muscle strength in her lower extremities and was diagnosed with late-onset paralysis resulting from a T12 vertebra fracture. Her muscle strength in the lower extremities was significantly decreased (manual muscle test [MMT]: iliopsoas 3/3, quadriceps 3/3, tibialis anterior 1/1, extensor hallucis longus 1/1, gastrocnemius 2/2). CT images showed a severe collapse of the T12 vertebra (Figure 5(a)), and a bone fragment of the T12 posterior wall displaced into the spinal canal (Figure 5(b)). MRI showed the collapsed T12 vertebra with T1–weighted image (WI) low and T2–WI partial high, and the displaced T12 posterior wall compressing the spinal cord (cauda equine) (Figures 5(b) and 5(d)).

**Figure 6:**
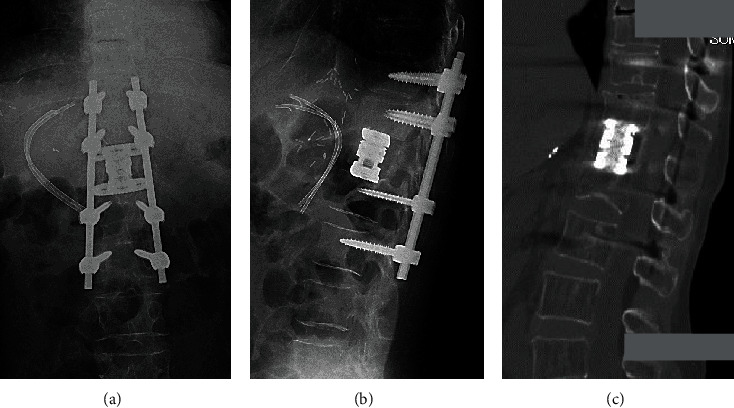
Postoperative radiographs and computed tomography (CT) images of the thoracolumbar spine of a 66-year-old woman with collapse of the 12^th^ thoracic (T12) vertebra. (a) anteroposterior view X-ray; (b) lateral view X-ray; (c) sagittal CT image two years following surgery.

The patient underwent posterior instrumentation surgery followed by T12 corpectomy using a wide-foot print expandable cage (X-core, NuVasive, Inc.) (Figures 6(a) and 6(b)). First, pedicle screws were inserted into the T10, T11, L1, and L2 vertebrae bilaterally, and a Titanium alloy rod was placed for alignment correction and distraction of the T12 vertebra collapse. Next, the patient underwent retropleural exposure for a T12 corpectomy using a wide-foot print expandable cage (X-core, NuVasive, Inc.) as previously reported [9]. Briefly, the patient was placed in the left lateral decubitus position with the skin incision along the left T10 rib. After removing a small segment (7 cm) of rib, the T12 vertebra was safely exposed through a retropleural approach without adhesion with the surrounding tissue. By using an expandable retractor (MaXcess®, NuVasive, Inc.), the T12 vertebral body replacement was performed by a wide-footprint expandable Ti cage (XCore®, NuVasive, Inc). Autograft (removed rib) with hydroxyapatite/type I collagen (HAp/Col) composite [14] was packed inside and outside the cage. The operation time was 360 minutes, and intraoperative blood loss was 133 mL. Postoperative intravenous antibiotic (Cefazolin: 1 g × 2/day) was administrated for seven days. Following surgery, no complications associated with the transplanted liver were identified.

No remarkable changes on the thoracolumbar radiograph, including vertebral fractures and/or mechanical complications, were identified during two years postoperatively. Sagittal reconstruction CT images taken two years following the spinal surgery revealed robust bridging bone inside and outside of the cage (Figure 6(c)). Her muscle strength in the lower extremities was significantly improved, and she could walk without a cane 2.5 years following spinal surgery.

## 3. Discussion

We have reported the first two cases of the clinical course, radiologic features, and surgical outcome of lateral access spine surgery following liver transplantation surgery.

The more significant part of the liver is situated under the cover of the ribs, costal cartilages, and diaphragm. The average highest point of the upper surface of the liver is located at about the level of the T9 vertebra, while the lowest average point is at the L2 vertebra level. For the first step of liver transplantation recipient surgery, the right and left lobes of the liver are mobilized from the diaphragm. Next, the vena cava is exposed, and then fully mobilized from the retroperitoneum. The portal vein, hepatic artery, and bile duct are then exposed. After dissecting these blood vessels and the bile duct, the diseased liver can be removed. For the next step, these blood vessels and the bile duct of the recipient are anatomized to those of the donor tissue [16]. Therefore, there is a possibility that tissue adhesion in the retroperitoneum can occur around the vena cava at the T9 to L2 level. Our report demonstrates that lateral access spine surgery for middle and/or lower lumbar levels can be safely performed for patients following liver transplantation. For thoracolumbar lesions (from T10 to L2), a retroperitoneal or retropleural lateral approach can be safely performed from the left side. However, a right side approach to a thoracolumbar lesion should be avoided because of tissue adhesion around the transplanted liver. In our two cases, splenectomy was simultaneously performed during the liver transplantation surgery. Nevertheless, a lateral approach to the thoracolumbar and lumbar lesion was safely performed without adhesion with the surrounding tissue. Importantly, the authors suggest that the planning of spine surgery should be determined after consulting with the liver transplantation team.

In 1998, Testa et al. reported the type and incidence of surgical procedures in 409 patients who had previously undergone liver transplantation [17]. Among these, orthopedic surgeries, including spine surgery (two patients), were performed in 47 patients, and their complication rate was 10.7%. The authors concluded that most surgical procedures could be safely performed without an increased incidence of complication. Shaikh et al. reported eighteen cases who underwent spinal fusion surgery following organ transplantation, including liver transplantation, between 1997 and 2008 [3]. They revealed that there was one death immediately after spine surgery and one Graft-versus-host disease (GVHD). Among thirteen patients of these eighteen who underwent radiographic evaluation, 12 patients showed radiographic bone fusion. The authors concluded that spinal fusion surgery could be performed successfully and safely after organ transplantation; however, the complication rate was higher than in the nontransplant group. More recently, Kimura et al. reported one case of a spinal paralysis patient who was successfully treated with total *en bloc spondylectomy* (TES) for T7 metastasis after living donor liver transplantation for hepatocellular carcinoma [11]. Any complications related to the transplanted liver were not reported. Following spinal surgery, the patient could walk with no evidence of recurrence and without liver failure.

These previous reports suggest that spinal surgery can be safely performed for patients following liver transplantation; however, utmost attention should be paid for postoperative complications. In our cases, a severe liver disorder did occur at an early stage following spine surgery in case 1. The authors have recognized that the cause of the liver disorder was acetaminophen-induced hepatotoxicity [18]. At present, no evidence-based guidelines exist on the use of analgesic drugs for liver transplantation patients; however, intravenous administration of fentanyl or oral administration of tramadol can be recommended for postoperative pain management of liver transplantation recipients [19, 20].

It has been reported that a rapid decrease in bone mineral density (BMD) occurs in the early stage (from 3 to 6 months) following liver transplantation: this is also associated with the occurrence of osteoporotic vertebral fractures (OVFs) [21–23]. Osteoporotic changes following liver transplantation are considered to be associated with multiple factors, including hepatic osteodystrophy, malnutrition, immobility, hypogonadism, and the use of immunosuppressants [21, 23]. Among immunosuppressants, calcineurin inhibitors, such as cyclosporine and tacrolimus, inhibit the differentiation and proliferation of T-cells and show potent immunosuppressive effects. Importantly, it has been reported that the risk of fractures increases in patients treated with calcineurin inhibitors because of rapid bone loss following transplantation [5]. In our cases, the patient (case 2) who had used tacrolimus (1.5 mg/day) for 67 months showed a significant collapse of the T12 vertebra following an OVF. This suggests that prevention and management of bone loss in the liver transplant recipient are of great importance to prevent OVFs in their posttransplant follow-up.

Liver transplantation recipients also have a risk of postoperative infection because of the induction of immunosuppressive therapy [4]. Although little has been reported in spinal instrumentation surgery following organ transplantation, a previous study has shown that patients who had joint replacement surgery following organ transplantation, including liver transplantation, have a high risk of infection [24]. According to a previous study that evaluated the clinical results of spinal fusion surgery following organ transplantation in 18 patients [3], no deep infection associated with the spinal instrument was reported. Although no postoperative infection was identified in our two cases, the authors consider that a careful follow-up for the occurrence of a postoperative infection should be performed.

In conclusion, a retroperitoneal transpsoas lateral approach for middle and/or lower lumbar levels can be safely performed for patients following liver transplantation without adhesion with the surrounding tissue. For thoracolumbar lesions, a retroperitoneal or retropleural lateral approach can be safely performed from the left side. The two cases presented here suggest that lateral access spine surgery can be successfully performed for patients following liver transplantation. However, any complications related to the function of the liver graft should be monitored following the spine surgery.

Minimum invasive surgery (MIS), including lateral access surgery, has an advantage for reducing the incidence of intra- and postoperative complications, including bleeding and/or infection for the liver transplant recipient. However, sufficient attention should be paid to the postoperative complications, especially a deep incisional surgical site infection, that need salvage surgery. Therefore, the decision of surgical procedure should be made after thorough consultation with a transplantation team about the possible complications of lateral access surgeries.

## Figures and Tables

**Table 1:** Patient's characteristics.

Case	#1	#2
Age	49	66
Gender	Female	Female
Diagnosis of liver disorder	Acute-on-chronic liver failure	Hepatocellular carcinoma
Immunosuppressant drug	Cyclosporine	Tacrolimus hydrate
Spinal disorder	Degenerative lumbar disease	T12 vertebral fracture
Spine surgery	LLIF (L3/4, L4/5), PLIF (L5/S1), L3-S1 instrumentation	T12 corpectomy, T10-L2 instrumentation
Follow-up	27 months	31 months
Complication	Transplanted liver dysfunction	None

LLIF: lateral lumbar interbody fusion; PLIF: posterior lumbar interbody fusion.

## Data Availability

This is a case report of two patients, to protect privacy and respect confidentiality; none of the raw data has been made available in any public repository. The original reports, laboratory data, images, and clinic records are retained as per normal procedure within the medical records of our institution.

## Supplementary Materials

Supplementary Materials The CARE guidelines (for CAse REports) were used for drafting the manuscript. The CARE checklist was uploaded.
